# Non-Steroidal Anti-Inflammatory Drugs in the Aquatic Environment and Bivalves: The State of the Art

**DOI:** 10.3390/toxics12060415

**Published:** 2024-06-05

**Authors:** Pedro Pires, André M. P. T. Pereira, Angelina Pena, Liliana J. G. Silva

**Affiliations:** LAQV, REQUIMTE, Laboratory of Bromatology and Pharmacognosy, Faculty of Pharmacy, University of Coimbra, Polo III, Azinhaga de Sta Comba, 3000-548 Coimbra, Portugalapena@ff.uc.pt (A.P.)

**Keywords:** bivalves, environmental bioindicators, pharmaceuticals, non-steroidal anti-inflammatory pharmaceuticals, consumption, occurrence, analytical methodologies

## Abstract

In recent years, contaminants of emerging concern have been reported in several environmental matrices due to advances in analytical methodologies. These anthropogenic micropollutants are detected at residual levels, representing an ecotoxicological threat to aquatic ecosystems. In particular, the pharmacotherapeutic group of non-steroidal anti-inflammatories (NSAIDs) is one of the most prescribed and used, as well as one of the most frequently detected in the aquatic environment. Bivalves have several benefits as a foodstuff, and also as an environment bioindicator species. Therefore, they are regarded as an ideal tool to assess this issue from both ecotoxicological and food safety perspectives. Thus, the control of these residues in bivalves is extremely important to safeguard environmental health, also ensuring food safety and public health. This paper aims to review NSAIDs in bivalves, observing their consumption, physicochemical characteristics, and mechanisms of action; their environmental occurrence in the aquatic environment and aquatic biota; and their effects on the ecosystem and the existent legal framework. A review of the analytical methodologies for the determination of NSAIDs in bivalves is also presented.

## 1. Introduction

Since the late 1970s, pharmaceutical environmental pollution has emerged as a significant threat to both human health and the biosphere, and it is now widely recognized as a global challenge [1,2].

Pharmaceutical products play a fundamental role in our society by allowing for the prevention, control, and treatment of various diseases, resulting in a better quality and life expectancy. These substances are designed to cause, in low concentrations, a specific and beneficial biological effect both in animal and human health care [1,3]. The available data on drug consumption are often inadequate or insufficient to estimate the true consumption patterns of each country. Moreover, many pharmaceutical products are over-the-counter medicines, leading to greater uncertainty regarding their real consumption [2].

Non-steroidal anti-inflammatory drugs (NSAIDs) are extensively used on a global scale to treat a diverse range of inflammatory conditions, fever, and both acute and chronic pain. After human excretion, NSAIDs present quite variable average removal rates in wastewater treatment plants (WWTPs), ranging between 77% and up to 96% (paracetamol (PAR)), with the exception of diclofenac (DIC) (34%) [4], and are constantly introduced into aquatic environments, where they can remain for long periods [5]. Their presence is considered to be a threat to ecosystems and to inhabiting non-target organisms and, ultimately, to public health [6]. The effects of these pollutants, some associated with toxicity, mutagenicity, and carcinogenicity, on aquatic biota and human health remain scarcely understood. Thus, further knowledge regarding their accumulation and metabolization by aquatic biota is mandatory.

NSAIDs are among the most widely consumed pharmacological groups worldwide in both human and veterinary medicine [7], and this widespread consumption is associated with their presence in the aquatic environment. Several studies have reported the environmental presence of different drugs from this therapeutical group, placing them among the most commonly detected drugs in aquatic environments [8].

Biomonitoring is a valuable tool to determine biota exposure to hazardous pollutants, allowing us to assess the quality of the aquatic environment and point out threshold levels that might be introduced into the human food chain [9]. Bivalves are sessile filter feeders that bioconcentrate chemicals, making them ideal organisms to use as indicators of environmental health hazards. Moreover, bivalves are key components of coastal and estuarine ecosystems. They are usually biomass dominant and highly productive, play a central role in the food web linking primary producers and epibenthic consumers, and provide essential ecosystem services [10]. They are also economically valuable as a food resource, and are harvested for human consumption for centuries and, more recently, produced in aquaculture to supply the growing consumption demand. Nonetheless, much of bivalve harvesting and production is confined to estuaries and coastal areas, where sources of human sewage pollution proliferate and where these filter-feeding animals bioconcentrate anthropogenic contaminants [11]. Therefore, in addition to the possible toxic effect on bivalves, bioconcentration of these chemical contaminants by these aquatic organisms may raise special concerns towards the protection of public health and consumer confidence in shellfish products [9].

Hence, a better understanding of the fate of these chemical substances is of great importance to trigger further actions to prevent adverse impacts both on environmental and human health. Thus, the present work aims to bring together the information retrieved from NSAID studies in the aquatic environment and their occurrence in bivalves over the last 20 years. Aiming to improve environmental and human health, raising awareness for environment policies in Europe, a critical review of the available information was performed, also taking into consideration the physicochemical characteristics and mechanisms of action of NSAIDs; their effects on bivalves; the legal framework; and the available analytical methodologies.

## 2. Methodology

The available scientific literature was searched using the Thomson Reuters ISI Web of Knowledge, PubMed, Science Direct, and Google Scholar databases.

Combinations of at least two of the following keywords were used: “Bivalves”; “Bioindicators”; “Pharmaceuticals”; “Non-steroidal anti-inflammatory pharmaceuticals”; “Consumption”; “Occurrence”; “Analytical methodologies”. The inclusion criteria for the selected studies were the determination of NSAIDs and/or metabolites in environmental and bivalve samples, and full access to the study; studies not reporting original data were excluded. Overall, the literature search identified a total of 86 studies published between 1999 and 2023.

## 3. Bivalves

Bivalvia, or bivalves, are a class of the phylum Mollusca (second largest phylum in the animal kingdom), exclusively aquatic organisms (mostly marine), which have a common characteristic: a shell formed by two valves that extend dorsally and protect the mollusks. Major groups in this class include mussels, clams, cockles, scallops, and oysters [12].

Most species of bivalves are considered to be ecologically important [13]. They are abundant and are widely distributed across different habitats, and are sessile species with a long lifespan, which allows them to be easily captured [14].

Bivalve mollusks breathe and feed by filtering large amounts of water and are highly efficient filter feeders. However, bivalves cannot selectively filter food particles (mainly phytoplankton and zooplankton) present in the water column, which leads them to bioaccumulate and bioconcentrate various types of substances also present in the water column in their tissues, among which various types of contaminants are found such as drugs and pharmacologically active compounds [15].

### 3.1. Bivalves as Foodstuff

Bivalves are a widely consumed food product with high commercial and nutritional value, having been part of the human diet for thousands of years.

The consumption of this food is associated with multiple benefits, such as improved neuronal development and a reduced incidence of cardiovascular diseases [16]. These benefits are due to its nutritional composition, particularly its high protein content; vital nutrients such as vitamin B12; minerals such as potassium (K), sodium (Na), and phosphorus (P); and essential polyunsaturated fatty acids, including omega-3 [17,18].

Several species of bivalves are part of our diet, including mussels such as the common mussel (*Mytilus edulis*) and the Mediterranean mussel (*Mytilus galloprovincialis*), oysters such as the Pacific oyster (*Crassostrea gigas*), and clams such as the white clam (*Spisula solida*), the “donkey’s foot” (*Chamelea gallina*), and the clam (*Ruditapes decussatus*). Other highly consumed species include scallops (*Pecten maximus*) and cockles (*Cerastoderma edule*). All of these species are highly consumed worldwide and have high commercial value. Despite their benefits, these seafoods can pose a high risk to public health in terms of food safety and are considered to be vectors of foodborne diseases [15].

Since bivalves feed and breathe by filtration, they accumulate and concentrate various substances present in the water column in their tissues, exposing humans to higher concentrations of these substances. These substances include biological contaminants of natural origin, such as pathogenic bacteria and viruses, (ex: *Escherichia coli* and norovirus) [19,20], marine biotoxins, and also different chemical contaminants such as polycyclic aromatic hydrocarbons (PAHs), dioxins, polychlorinated biphenyls (PCBs), and pharmaceuticals, including NSAIDs [11,21,22].

The impact on public health can be direct, resulting from the consumption of shellfish contaminated with pharmaceuticals at high concentrations, potentially causing allergic reactions. Additionally, low concentrations can promote chronic toxicity, particularly due to their potential effects as endocrine disruptors. Indirect impacts include the possibility of developing antimicrobial resistance, among other issues. [22]. The problem may persist or even increase after cooking, specifically in mussels [23].

### 3.2. Bivalves as Bioindicators of Environmental Contamination

A bioindicator is defined as an organism or microorganism that is used to monitor natural ecosystems, as well as possible changes in them [24]. As mentioned above, bivalves mainly assimilate, various types of substances present in the water column [23] in the digestive gland and gills, among which contaminants, such as drugs, are bioaccumulated and bioconcentrated by these mollusks. This characteristic, combined with their wide distribution and high population density, as well as their high tolerance to different environmental conditions, makes bivalves excellent bioindicators [25], namely of drugs [26].

In the case of aquatic organisms such as bivalves, the accumulation of a given substance is called bioconcentration and depends on the physical–chemical characteristics of the substance. The bioconcentration factor (BCF) is the ratio between the concentration of the substance in the body and the concentration in the water. Biomagnification consists of transfer of accumulated xenobiotic substances along the successive trophic levels of the food chain [27].

Previous studies on bivalves have shown a low bioconcentration potential at higher or less environmentally realistic doses, possibly due to a reduction in filtration rate under more stressful conditions. At environmentally realistic concentrations, most drugs were detected in their tissues without clear time- or concentration-dependent trends, but with notable species-specific differences. These differences are likely due to variations in biotransformation and/or excretion capacities [25].

## 4. Non-Steroidal Anti-Inflammatory Drugs (NSAIDs)

NSAIDs are a highly diverse pharmacological group with varied chemical characteristics but with common therapeutic properties. Despite their proven effectiveness, they have some adverse effects, namely at the gastrointestinal, cardiac, and renal systems [27].

The first NSAID was developed in 1897 by Felix Hoffman (at the German company Bayer), who formulated acetylsalicylic acid (AAS), commonly known as aspirin [28]. Since then, several NSAIDs have been produced, becoming one of the most prescribed and consumed pharmacological substances in modern medicine. Ibuprofen (IBU), DIC, naproxen (NAP), ketoprofen (KET), and phenazone (PHE) stand out as some of the most prevalent.

The use of NSAIDs is generally associated with the treatment of various inflammatory conditions including chronic diseases such as rheumatism and osteoarthritis [29], and also in the treatment of pain and other conditions such as fever, headaches, and other diseases due to their anti-inflammatory, analgesic, and antipyretic properties [7]. AAS, besides its use as an NSAID, is also consumed in low doses as a platelet aggregation inhibitor, reducing the risks associated with cardiovascular diseases.

Due to their pharmacological properties, NSAIDs are one of the most consumed pharmacological groups worldwide [7] and, consequently, are widely reported in the aquatic environment [8]. In addition to being highly prescribed, many of these drugs are over-the-counter medicines, with greater ease of access, which makes their consumption even higher than estimated.

### 4.1. Physical–Chemical Characteristics

The molecular formulas, as well the most relevant physical–chemical characteristics, of some of the most studied and detected NSAIDs in bivalves are shown in Table 1.

As one can observe, NSAID log Kow values vary between 0.38 and 5.12. As for pKa, the values oscillate between 1.4 and 9.38. Paracetamol, not having anti-inflammatory properties and not considered to be an NSAID by many authors, has a pKa of 9.38 [30].

### 4.2. Mechanisms of Action

In 1971, John Vane, a British pharmacist, discovered the mechanisms of action of salicylic acid (SA) and other NSAIDs [31]. There are two isoforms of the cyclooxygenase enzyme (COX), cyclooxygenase 1 (COX-1) and cyclooxygenase 2 (COX-2). These enzymatic complexes catalyze the production of prostaglandins (PGs) from arachidonic acid, which is present in the cell membranes of phospholipids. PGs are prostanoids (synthesized by COX) that can present several forms (PG H, PG E, PG D, and thromboxane A) and are involved in and mediate several physiological and pathological processes, such as fever, pain, and inflammation, as well as preventing blood clot formation, the development of cardiovascular pathologies, and osteoporosis. On the other hand, PG G2 leads to the formation of thromboxane A (TXA), which is involved in platelet aggregation [32,33].

In general, NSAIDs’ mode of action involves inhibiting PG biosynthesis, preventing the binding of arachidonic acid to the COX enzymatic complex (COX-1 and/or COX-2) in the central and peripheral nervous system [7]. This translates into NSAIDs’ antipyretic, analgesic, and anti-inflammatory properties [34].

As already mentioned, PAR, or acetaminophen, does not have anti-inflammatory activity, and it is recognized only for its analgesic and antipyretic properties. Its mode of action differs from NSAIDs as it acts preferentially on COX-2 and COX-3 [28].

More recent NSAID formulations have COX-2-selective inhibition as their mode of action [29]. This new approach overcame the COX-1 inhibition that was identified as one of the main factors responsible for the side effects of NSAIDs [35].

## 5. Environmental Occurrence of NSAIDs

The active substances belonging to this pharmacological group are among the most detected, both in the aquatic environment and in the aquatic biota [36]. Below we summarize the occurrence data on some of the most relevant NSAIDs in different aquatic bodies and bivalves, respectively.

### 5.1. Aquatic Environment

Considering the different sources of NSAIDs and their constant release into the aquatic environment, they have been detected and quantified in various water bodies such as effluents and effluents from WWTPs, effluents and effluents from industries and hospitals, groundwater (aquifers), surface water (marine waters, rivers, lakes, swamps, estuaries), and even in drinking water.

The values at which they were detected in these water bodies vary between ng/L and µg/L [26], and their detection frequencies depend on the matrix. Generally, they follow a decreasing order of concentration: industrial effluents > hospital effluents > WWTP influents > WWTP effluents > surface water > groundwater > drinking water [2].

Regarding WWTPs, there is usually a reduction in the concentration of drugs present in the effluents, when compared to their influents. As seen in Paíga et al. [37], there was a decrease in the maximum concentration of PAR from 615.135 µg/L to 4.909 µg/L and of IBU from 24.505 µg/L to 3.304 µg/L, which translates into removal efficiencies in the WWTP of about 99.60%. and 86.52%, respectively.

As shown in Table 2, NSAIDs are detected in drinking water at concentrations well below their therapeutic doses with values between 0.055 µg/L for NAP [38] and 1.35 µg/L for IBU [39].

### 5.2. Aquatic Biota

The ubiquitous and concerning presence of NSAIDs in aquatic bodies cannot be ignored. As a result, these substances end up accumulating in aquatic biota, causing harm to aquatic ecosystems.

In contrast to studies on environmental waters, research on the presence of NSAIDs in aquatic biota remains limited. Nonetheless, interest in studying the occurrence of drugs in bivalves is increasing, both due to their high human consumption as well as due to the fact that these species are excellent bioindicators of aquatic pollution [52]. Miller et al. [53] reported only 43 publications (until 2018) relating to the occurrence of drugs in aquatic biota, 29 relating to fish and 18 to invertebrates. In these publications, 200 drugs were quantified, of which 14% belong to NSAIDs whose residues in invertebrates were highlighted as the class with the highest concentrations (median of 20.50 ng/g). Among the NSAIDs, the most detected drugs were DIC and IBU with medians of 15 ng/g and 83.65 ng/g, respectively.

Table 3 summarizes the occurrence of NSAIDs in bivalve species found in the scientific literature.

As seen in Table 3, concentrations varied between undetected and unquantified values, to some hundred ng/g, which demonstrates a lower presence in bivalves when compared to aquatic bodies. The values obtained by Wolecki et al. [61] for DIC (560 ± 130 ng/g), IBU (730 ± 290 ng/g), and NAP (560 ± 130 ng/g) stand out as the highest concentrations found. Despite the apparent low concentrations of the revised values, data point out potential adverse effects on these organisms [63].

## 6. Effects of NSAIDs on Bivalves

To evaluate the toxic effects of drugs, specifically NSAIDs, on different organisms, specific toxicological tests are needed to evaluate both acute and chronic effects. In acute toxicity tests, the occurrence of mortality is recorded, while in chronic toxicity tests, other factors are evaluated, such as the impact on reproduction and growth rates [64].

In the aquatic environment, fish are the most frequently contaminated organisms, and information about the effects resulting from contamination of other organisms, such as invertebrates and aquatic plants, is still limited due to structural differences that prevent an extrapolation of results [64].

The occurrence of adverse effects on aquatic biota resulting from exposure to pharmaceuticals, which can cross biological membranes and reach specific cells and tissues, is consequential. Chronic toxicity is also associated with potential bioaccumulation, representing an environmental risk to aquatic biota [8,65].

Most anti-inflammatory drugs cause a non-specific inhibition of prostaglandins, which means that they have the potential to affect any normal physiological functions mediated by prostaglandins [66]. In a recent review, DIC, IBU, and PAR were reported to bioconcentrate in marine bivalves, showing recognized effects at various life stages, with long-term exposure primarily targeting immune responses [25].

Some studies have already been carried out to evaluate the impact of NSAIDs, namely AS and DIC, on bivalves and, more specifically, on mussels. In these studies, changes in redox balance, antioxidant mechanisms, and metabolism, as well as an increase in oxidative stress, were observed in mussels, resulting from exposure to these contaminants. These changes can compromise the growth and reproduction of these species [67]. According to Wolecki et al. [61], PAR induces some genotoxicity in the mussel *Dreissena polymorpha*, but without causing damage to its DNA. In the same study, it was concluded that both DIC and IBU have cytotoxic effects on this species of mussel. Another study confirmed the effects of DIC on the embryonic development of *Mytilus galloprovincialis*, observing malformations in the species’ shells [68].

Recently, the single and combined effects of caffeine (CAF) and SA were studied on the mussel *Mytilus galloprovincialis,* which was exposed for 12 days. It was observed that CAF, when administered individually at environmentally relevant doses, may induce oxidative stress in the digestive gland of mussels. In contrast, SA alone may reduce cellular ROS production by affecting mitochondrial activity. Consequently, transcription modulation, along with changes in enzymatic activity, constitutes part of the early defense response activated to mitigate CAF-induced oxidative stress. Exposure to the CAF and SA mixture elicited significantly different transcriptional and biochemical responses compared to CAF alone, primarily due to the effects of SA [69].

Some ecotoxicological data also report that mixtures of polluting compounds (other contaminating chemical compounds such as pesticides) present different effects from those observed in isolated compounds due to the possible occurrence of additive effects. These possible effects must be taken into account when evaluating the ecotoxicology of drugs in bivalves [64].

In addition to the ecotoxicological effects that can impact the metabolism, physiology, and even reproduction of various aquatic organisms, the widespread presence of drugs raises concerns about human health. These issues relate to the potential for drug transfer into food, which can cause direct or indirect toxic effects through antibiotic-resistant bacteria [70].

## 7. Legal Framework

To protect humans and the environment from exposure to certain contaminants, the European Parliament and the European Commission (EC) have established regulations, setting maximum levels for some of these contaminants. Regarding food, regulation (EC) No. 1881/2006 of the European Commission [71] established maximum limits for some contaminants, which were recently amended by Commission Regulation (EU) 2022/1370 of 5 August 2022 [72].

However, over the last decades, there has been an increase in the levels of chemical compounds resulting from anthropogenic activity, such as drugs and other compounds [53], for which no maximum limits have been established [73].

As the use of these compounds cannot be avoided, it is essential to assess the environmental risk associated with their contamination in order to comply with the Water Framework Directive. This was introduced with Directive 2000/60/EC (https://eur-lex.europa.eu/legal-content/EN/TXT/?uri=celex%3A32000L0060, accessed on 18 April 2024) of the European Parliament and the Council, and it establishes a framework for community action in the field of water policy, aiming to have all member states guarantee the environmental quality of their aquatic bodies. Since then, it has been amended by several directives such as Directive 2008/105/EC (https://eur-lex.europa.eu/eli/dir/2008/105/oj, accessed on 18 April 2024), which was implemented to control the presence of organic and inorganic contaminants in surface waters and which established a list of 33 substances/groups of substances that should be monitored. This directive was later amended by Directive 2013/39/EU (https://eur-lex.europa.eu/eli/dir/2013/39/oj, accessed on 18 April 2024).

Directive 2013/39/EU (https://eur-lex.europa.eu/eli/dir/2013/39/oj, accessed on 18 April 2024). of 12 August 2013 updated the priority substances in the field of water policy and established a new watch list. This is a dynamic list, updated based on the persistence and concern associated with the presence of pharmaceuticals (and other contaminants) in water cycles, thus its validity is limited. Therefore, it is extremely important to identify and prioritize the most prevalent drugs so that they are monitored more frequently [5]. According to this directive, all member states must monitor the substances listed on the watch list in the indicated watercourses/monitoring stations. This data collection allows the EU to investigate the impacts that contaminants of emerging concern, including pharmaceuticals, and other substances have on the environment and to determine their legal priority [74]. In the case of Portugal, six monitoring stations have been identified, from which samples must be collected at least once a year to analyze the presence of these contaminants [4]. This directive also recommended that DIC, together with the hormones 17-beta-estradiol (E2) and 17-alpha-ethinyl estradiol (EE2), be included in the first watch list for the collection of monitoring data.

The first watch list was published with Implementing Decision (EU) 2015/495 https://eur-lex.europa.eu/eli/dec_impl/2015/495/oj, accessed on 18 April 2024) and, in addition to the three compounds mentioned above, also included the hormone estrogen (E1) and three antibiotics (erythromycin, azithromycin, and clarithromycin), among other contaminants. [74]. This decision was in turn revoked by Implementing Decisions (EU) 2018/840, (https://eur-lex.europa.eu/eli/dec_impl/2018/840/oj, accessed on 18 April 2024) 2020/1161 (https://eur-lex.europa.eu/eli/dec_impl/2020/1161/oj, accessed on 18 April 2024), and 2022/1307 (https://eur-lex.europa.eu/eli/dec_impl/2022/1307/oj, accessed on 18 April 2024). The actual list of pharmaceuticals is composed of antibiotics (ciprofloxacin, amoxicillin, sulfamethoxazole, and trimethoprim), antidepressants (venlafaxine and O-desmethylvenlafaxine), and antifungals (clotrimazole, fluconazole, and miconazole).

Recent studies support the re-inclusion of DIC on the European Union watch list and emphasize the need for further research on ibuprofen and paracetamol due to their demonstrated negative effects on marine bivalves when exposed at environmentally realistic concentrations under laboratory conditions [25].

## 8. Analytical Methodologies for the Determination of NSAIDs in Bivalves

Pharmaceutical analysis in complex matrices such as bivalves, requires a protocol that is well adapted to the specific physicochemical characteristics of the target drugs, namely NSAIDs, and to the matrix itself [75]. The analytical methodology must include efficient extraction and purification steps, as well as adequate sensitivity and specificity to detect and quantify the analytes in study [76].

Below is a compilation of sample preparation/extraction and purification methods, along with instrumental analysis techniques for the determination of NSAIDs. Additionally, it includes parameters associated with the efficiency of the methodology used, such as analytical limits and recovery percentages. 

### 8.1. Sample Preparation and Extraction

Sample preparation and extraction can be a long-lasting and extensive steps due to the complexity of the biotic matrices, and also to the physicochemical characteristics of the analytes [77,78]. Ideally, sample preparation should be fast, accurate, and precise while maintaining sample integrity. Moreover, the development of multiresidue extraction methods is a challenge because of the different physicochemical properties of the compounds [63].

Freeze-drying is frequently used in the pre-treatment of samples [53], which allows for the samples to be preserved frozen and “dry” under vacuum for prolonged storage periods [79].

One of the first steps in extraction is homogenization, which consists of forming a suspension or emulsion from solid tissues [80]. Tissue homogenization can be achieved by mechanical techniques using blenders, mills, agitators, vortex mixers, or ultrasonic equipment [53]. This step is crucial for extraction efficiency [76].

After homogenization, ethyl acetate, acetonitrile (ACN), acetone, and methanol (MeOH) are some of the organic solvents most used [53]. ACN is the most used extraction solvent alone [58] or in mixture with other solvents, which mostly comprise water [23,54,65,78,81], formic acid [55,82], and MeOH [70] (Table 4).

To effectively extract the analytes, one must choose the extraction technique that best suits the properties of the analyte and that most effectively removes the interferents. There are several techniques used to extract drugs from bivalves and other aquatic biota, as presented below.

#### 8.1.1. Pressurized Liquid Extraction

Also known as accelerated solvent extraction (ASE), pressurized liquid extraction (PLE) is one of the most used techniques, presenting some advantages, such as greater reproducibility, given its semi-automation, reduced use of solvents, and also lower time expenditure, processing multiple samples simultaneously [77,82]. The extraction takes place in a closed-flow system, which has an extraction cell and an oven where the extraction takes place. To achieve this, organic solvents are used at temperatures and pressures that can vary between 100 and 180 °C, and 1500 and 2000 psi. By increasing these two conditions, the solubility of the analytes increases and the viscosity of the solvents decreases; therefore, there is greater diffusion of the analytes into the solvent, which leads to an increase in the effectiveness of extraction of the analytes from the matrix [83].

Despite presenting some disadvantages such as a high equipment cost and the selectivity of the technique, it is widely used in the extraction of drugs, including NSAIDs, from bivalves, as can be seen in Table 4 [14,23,26,54,55,61,76,81].

#### 8.1.2. QuEChERS

QuEChERS, an acronym for Quick, Easy, Cheap, Effective, Rugged, and Safe, offers several advantages, such as ease of application, speed, and reduced cost, and is widely used in determining food contaminants [76]. This methodology was first introduced in 2003 [84] to extract pesticides from food matrices. It involves a microscale extraction, using ACN or other solvents mixed with salts to favor the separation between water and solvent, ligands, and buffers for the extraction of organic residues, including NSAIDs [53,58,65,76,78]. For instance, citrate was used by some authors following purification through the dispersive solid-phase extraction (d-SPE) technique [65,76].

ACN is the extraction solvent of choice as it generates fewer interferences compared to other solvents, such as acetone or ethyl acetate, and is more easily separated from water, allowing for higher extraction efficiencies [78,84]. Therefore, QuEChERS presents a good analyte recovery when compared to other techniques (Table 4) [76].

#### 8.1.3. Other Extraction Techniques

Other specialized extraction techniques have also been used, namely a micellar surfactant that allowed for very high recovery rates ranging from 97 ± 4% (NAP) to 106 ± 7% (IBU) [3].

Other non-specialized extraction techniques, such as ultrasound, centrifugation, and filtration mechanisms, have also been used after a simple solid–liquid extraction (SLE) using a specific organic solvent [22,70]. SLE is widely used, allowing for good recovery percentages that depend on which organic solvent or mixtures are used, with emphasis on the use of ACN and MeOH [82].

Another technique is solid–liquid extraction utilizing ultrasound (FUSLE), which involves a closed extractor equipped with a sonic probe that applies ultrasonic waves to the sample. It consists of a modification of the SLE that reduces the amount of organic solvent used and also the amount of sample required [82]. Other authors have also applied this procedure [75], obtaining recoveries between 93 and 112% for NSAIDs (PAR, KET, and DIC).

Table 4 presents the other extraction techniques previously mentioned.

### 8.2. Extract Purification

One of the major problems associated with determining analytes in complex matrices, such as bivalves, is the signal disturbance of the target analytes by the co-extracted interferents matrix due to their high polarity and similar chemical structures. Nonetheless, many methods described in the literature do not require a purification step after extraction [59].

Clean-up removes non-specific interferents, protecting the analytical system and also increasing the precision and accuracy of the method. Another objective of this step is to concentrate analytes to increase the sensitivity of the analytical methodology.

#### 8.2.1. Solid-Phase Extraction

This technique is widely used in drug analysis and has been used in several studies. The selectivity of the adsorbent depends on the type of interaction between the analyte and the adsorbent, which can be hydrophobic or hydrophilic. The SPE steps are directly dependent on the nature and physicochemical properties of the analyte, such as its polarity, its partition coefficient (log Kow), and its adsorption, and also on the properties of the matrix itself [85].

Table 5 describes the conditions under which the SPE was processed in some studies related to the purification of pharmaceuticals in bivalves, highlighting the prevalence of the use of MeOH and ultrapure water, in different proportions, throughout the three steps of SPE.

As a rule, the cartridges used are reversed-phase polymer columns, consisting of a polar adsorbing agent to remove lipids and other non-polar molecules [63]. Oasis HLB columns, which have a hydrophilic–lipophilic balance, were the most used in the reviewed bibliography due to their versatility and proven efficiency in pharmaceutical extraction [86]. Strata-X cartridges are also widely used [23,54,55,61] because although it is normally necessary to adjust their pH, they are considered to be quite effective in extracting drugs from different types of samples [86]. After SPE, the eluate is evaporated with nitrogen, avoiding the decomposition of the analytes of interest.

#### 8.2.2. Dispersive Solid-Phase Extraction

Dispersive solid-phase extraction (dSPE) is based on the dispersion of a solid adsorbent, mostly silica, added directly to the liquid samples during their extraction. This technique is widely used as it allows the extraction, isolation, clean-up, and even pre-concentration of analytes in complex matrices. It poses advantages due to its selectivity, robustness, and versatility, and also because steps such filtration are not necessary when using dSPE [87].

The use of this purification technique is often associated with QuEChERS extraction [58,65,76], but it can also be used as a clean-up strategy after techniques such as SLE [82].

### 8.3. Instrumental Analysis

Chromatography techniques are the most used instrumental methods in the determination of pharmaceuticals in biological matrices such as bivalves.

Advances in chromatographic technology have led to the development of highly sensitive and powerful instrumental analytical methods. These advancements have enabled the detection of emerging contaminants, such as pharmaceuticals, in complex environmental matrices [88] as well as new insights into environmental, biota, and human contamination.

Below, the chromatographic techniques most used to determine NSAIDs in bivalve samples, such as gas chromatography (GC), liquid chromatography (LC), and mass spectrometry (MS), are reviewed, and Table 6 compiles different analytical techniques and respective conditions for NSAID determination in bivalves.

Since GC is mainly used for the analysis of volatile and non-polar compounds, pharmaceutical determination requires a derivatization step in sample preparation [89]. The need for an extra step in GC-MS analysis due to the polarity and limited volatility that some compounds present makes LC the method of choice for determining these analytes [23]. However, some authors have used GC as an analytical method to determine five NSAIDs and three estrogens, obtaining recovery percentages that varied between 61 ± 11% (DIC) and 110 ± 9% (KET) [61]. Nowadays, UHPLC is frequently applied [14,22,55], justified by its greater speed, sensitivity, and efficiency [86].

In the reviewed bibliography, only reversed-phase columns of different dimensions were used (Table 6), mostly of C18 [3,14,22,23,26,54,70,75,76,81] or C8 [65] silica particles. C18 silica columns have a proven ability to effectively separate most ionizable pharmaceutical compounds [90], including NSAIDs [91].

Regarding the mobile-phase solvents, as seen in Table 6, the most used mobile-phase gradient elution solvents are H_2_O, MeOH, and ACN, both mixed in certain proportions and alone. ACN and MeOH are considered to be organic modifiers of choice and, since they are less polar, they are mixed with H_2_O [3,22,23,54,75,76], given NSAIDs’ required polarity [92].

Additives, acids, or bases are added to the mentioned solvents, and the choice of one or the other must be based on the characteristics of the analytes of interest, since these additives can condition the separation and sensitivity of the electrospray ionization (ESI) source [86]. Different additives were added to the mobile phases in the analysis of NSAIDs’ elution gradients. Among the reviewed articles, there was a preference for acidic mobile phases by adding formic acid, acetic acid, ethanoic acid, or methanoic acid in different percentages [3,65,75,76,78,81]. Other authors selected mobile phases in acidic and basic conditions by adding acidic and basic additives such as ammonia or ammonium acetate [14,22,26,58]. Fewer authors chose a basic mobile phase by adding 13 mM ammonium acetate to a H_2_O/ACN solution (80:20) [23,54].

The predominant use of acidic mobile phases is because the ionization of compounds with low pKa (as is the case with most NSAIDs whose pKa are mentioned in Table 1) is inhibited at acidic pHs. This inhibition causes the compounds to become less polar, increasing their interactions with the stationary phase and favoring their separation [93].

Regarding the detection of pharmaceuticals, in particular NSAIDs in bivalves, MS is undoubtedly the most prevalent method in the reviewed bibliography, as shown in Table 4 and Table 6. MS is widely used given its high sensitivity, selectivity, and specificity [94]. Ionization of analytes is mostly achieved through ESI, in both positive (+ESI) [55,65] or negative [78,81] modes. Only one author used heated electrospray ionization (HESI) as the ionization technique [55].

There are different mass analyzers, and the triple quadrupole (QqQ) is the most used in the determination of NSAIDs in bivalves (Table 6), followed by the ion trap mass analyzer [23,54] and the linear quadrupole IT (QqLIT) [14,26].

### 8.4. Analytical Performance

The latest EU regulation, 808/2021, on the performance of analytical methods to detect residues of pharmacologically active substances used in food-producing animals [95] defines several analytical parameters for analytical performance: specificity, selectivity, precision, recovery, ruggedness, linearity, and the decision limits CCα, LoD, and LoQ.

Regarding the reviewed bibliography, namely the linearity of the analytical response, we highlight the correlation coefficient (R^2^) of 0.99, obtained for NSAIDs in marine mussels [23,81].

Regarding the LODs, the results range between 0.01 ng/g (PRO-PHE) [14,26] and 50 ng/g (IBU and KET) [81]. As for the LOQs, they vary between 0.02 ng/g (PRO-PHE) [14,26] and 29 ng/g (DIC) [23,54]. The precisions obtained for the different NSAIDs vary between 2.6% and 18%. These precision values are considered to be acceptable as they are less than 20%.

Table 4 shows the recovery percentages obtained for each NSAID, and one can observe a large discrepancy between the reviewed studies. These range between 34.5% (for PHE) [14,26] and 112% (for PAR) [75]. As a rule, recovery percentages between 70 and 120% are considered to be acceptable.

## 9. Final Remarks

Bivalves, as filter-feeder organisms, are highly effective at bioaccumulating and bioconcentrating environmental contaminants, including pharmaceuticals. NSAIDs, in particular, are notable due to their widespread consumption, environmental ubiquity, and incomplete removal by wastewater treatment plants (WWTPs), one of the primary sources of pharmaceutical contamination in aquatic environments. Hence, NSAIDs have been identified in various aquatic environments and bivalve species. These substances have the potential to induce both acute and chronic ecotoxicological effects on bivalves, thereby negatively affecting their metabolism and physiology. Additionally, it is crucial to assess whether the consumption of bivalves could have adverse effects on human health.

It is of utmost importance to develop robust and reliable analytical methods, as well as to harmonize them into well-defined guidelines, so that the occurrence and extent of this problem can be assessed, both from an ecotoxicological and food safety perspective. It is urgent to counteract this trend of aquatic environmental pollution with pharmaceutical compounds, namely by improving WWTPs treatments and, consequently, reducing the toxic effects inherent to both ecosystems and humans. It is also crucial to carry out new studies on the effects of culinary treatments on these substances, original compounds, and corresponding metabolites to understand the real human risk. This will ensure that any potential health risks are identified and that appropriate measures are taken to mitigate them and guarantee food safety.

## Figures and Tables

**Table 1:** Physical–chemical characteristics of the main NSAIDs.

Pharmaceutical	CAS Number	Structure and Molecular Formula	Molecular Weight	Log Kow	pKa	Solubility (mg mL^−1^)
Diclofenac	15307-86-5	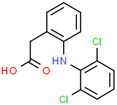 C_14_H_11_C_l2_NO_2_	296.1	4.51	4.15	2.37 ^a^
4′-Hydroxydiclofenac ^b^	64118-84-9	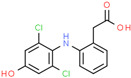 C_14_H_11_C_l2_NO_3_	312.1	3.18	_	17.9
Ibuprofen	15687-27-1	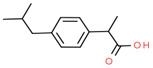 C_13_H_18_O_2_	206.28	3.97	4.91	21 ^a^
Paracetamol ^c^	103-90-2	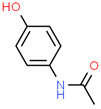 C_8_H_9_NO_2_	151.16	0.91	9.38	14 ^a^
Naproxen	22204-53-1	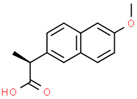 C_14_H_14_O_3_	230.26	3.18	4.15	15.9 ^a^
Ketoprofen	22071-15-4	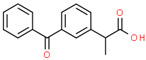 C_16_H_14_O_3_	254.28	3.12	4.45	51 ^a^
Phenazone	60-80-0	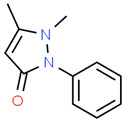 C_11_H_12_N_2_O	188.23	0.38	1.4	51,900 ^a^
Acetylsalicylic acid	50-78-2	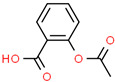 C_9_H_8_O_4_	180.6	1.18	3.5	4600 ^a^
Salicylic acid ^d^	69-72-7	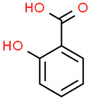 C_7_H_6_O_3_	138.12	2.26	2.97	2240 ^a^
Mefenamic acid	61-68-7	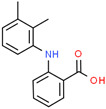 C_15_H_15_NO_2_	241.28	5.12	4.2	20
Piroxicam	36322-90-4	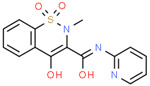 C_15_H_13_N_3_O_4_S	331.3	3.06	6.3	23

^a^—solubility at 25 °C; ^b^—DIC metabolite; ^c^—despite inclusion in this table, PAR does not have anti-inflammatory properties and is not considered to be an NSAID by many authors; ^d^—AAS metabolite.

**Table 2:** Occurrence of NSAIDs in different water bodies.

Aquatic Body	NSAIDs	Level (µg/L)	Local	Reference
Pharmaceutical industry effluent	PAR	12–64	Lahore, Pakistan	[40]
	NAP	215–464		
	DIC	252–836		
	IBU	703–1673		
	PAR	461	Ontario, Canada	[41]
	COD	49.2		
	IBU	344		
	NAP	253		
Hospital effluent	KET	<LOQ–0.199	Coimbra, Portugal	[42]
	NAP	0.0454–6.042		
	IBU	0.32–5.815		
	PAR	0.0130–0.0589		
	AS	0.383–2.817		
	DIC	<LOQ–0.189		
	PHE	0.0605–0.271		
	PRO-PHE	<LOD–0.0017		
	PIR	n.d.–0.0512		
	COD	0.0081–2.837		
Wastewater influent	DIC	0.46–6.5	Lisbon, Portugal	[43]
	KET	n.d.–1.7		
	IBU	8–53		
	NAP	n.d.–38		
	PAR	0.741–8.556	Tuscany, Italy	[44]
	KET	0.247–3.511		
	DIC	1.038–3.429		
	PAR	615.135 ^a^	Lisbon, Portugal	[37]
	IBU	24.505 ^a^		
	DIC	0.165 ^a^		
	NAP	3.245 ^a^		
	AS	61.259 ^a^		
	KET	0.147 ^a^		
Wastewater effluent	DIC	0.05–4.2	Leiria, Portugal	[43]
	KET	n.d.–0.72		
	IBU	n.d.		
	NAP	n.d.–3.3		
	PAR	<LOD–0.444	Toscana, Itália	[44]
	KET	0.031–0.512		
	DIC	0.811–4.882		
	PAR	4.909 ^a^	Lisbon, Portugal	[37]
	IBU	3.304 ^a^		
	DIC	0.724 ^a^		
	NAP	0.270 ^a^		
	AS	0.296 ^a^		
	KET	0.233 ^a^		
Groundwater	KET	2.886 ^a^	Europe	[45]
	IBU	0.395 ^a^		
	DIC	0.024 ^a^		
	NAP	0.0276	Serbia (Novi Sad, Zrenjanin, Bečej, Vrbas and Obrenovac)	[46]
	IBU	0.092		
	AS	<LOQ–0.0025		
	PHE	0.0234		
	PRO-PHE	<LOQ–0.0248		
	PAR	0.0009–1.036	Taiwan	[47]
	IBU	0.0174–0.837		
	NAP	0.128		
	KET	n.d.		
	DIC	0.0021–0.032		
Surface water	PAR	0.527 ^a^	Leiria, Portugal	[37]
	IBU	1.317 ^a^		
	DIC	0.038 ^a^		
	NAP	0.260 ^a^		
	AS	0.294 ^a^		
	KET	0.0753 ^a^		
Surface water	NAP	0.101 ^a^	Spain	[48]
	DIC	0.26 ^a^		
	4-OH-DIC	0.0482 ^a^		
	MA	n.d.		
	PAR	0.712		
	PHE	0.0375		
Seawater	PAR	0.584 ^a^	Atlantic ocean North Portuguese coast	[49]
	AS	0.0053 ^a^		
	DIC	0.241 ^a^		
	IBU	0.222 ^a^		
	NAP	0.178		
	KET	0.0897 ^a^		
Surface water	KET	0.045	Serbia (Novi Sad, Zrenjanin, Bečej, Vrbas and Obrenovac)	[46]
	NAP	<LOQ–0.0742		
	IBU	<LOQ–0.346		
	DIC	<LOQ–0.324		
	PHE	0.0125		
	COD	0.0073		
Drinking water	AS	0.2012	Spain	[50]
	DIC	0.140 ^b^	Stockholm, Sweden	[51]
	IBU	1.35 ^b^	Germany	[39]
	NAP	0.055	Queensland, Australia	[38]
	PAR	0.211 ^b^	Germany	[39]
	PHE	0.4 ^b^	Germany	[39]

4-OH-DIC—4′-hydroxydiclofenac; AS—salicylic acid; COD—codeine; DIC—diclofenac; IBU—ibuprofen; KET—ketoprofen; n.d.—not detected; LOD—limit of detection; LOQ—limit of quantification; MA—mefenamic acid; NAP—naproxen; PAR—paracetamol; PHE—phenazone; PRO-PHE—propyphenazone; ^a^ highest value detected; ^b^ maximum value.

**Table 3:** Occurrence of NSAIDs, in different locations, in several species of bivalves.

NSAIDs	Species	Level (ng/g)	Local	Reference
Mefenamic acid	*Mytilus edulis*	n.d. < 23	Ireland	[54]
Salicylic acid	*Mytilus edulis*	≤490	Belgium	[55]
Ketoprofen	*Mytilus galloprovincialis*	<LOQ	Adriatic Sea	[56]
	*Anomalocardia brasiliana*	0.53	Brazil	[57]
	*Mytilus edulis*	0.29	Brazil	[57]
Diclofenac	*Mytilus edulis*	n.d.	Ireland	[54]
	*Mytilus galloprovincialis*	<LOD–16.11	Adriatic Sea	[56]
	*Ruditapes decussatus*	1.1–6.2	Portugal	[58]
	*Mytilus spp*	0.5–4.5	Portugal	[59]
	*Corbiculidae*	1.2–31	China	[60]
	*Mytilus edulis trossulus*	560 ± 130	Adriatic Sea	[61]
	*Anomalocardia brasiliana*	<LOQ	Brazil	[57]
	*Mytilus edulis*	0.81	Brazil	[57]
Phenazone	*Crassostrea gigas*; *Chamelea gallina*; *Mytilus galloprovincialis*	<LOQ<LOD<LOD	Ebro Delta, Spain	[26]
Ibuprofen	*Geukensia demissa*	<LOQ	San Francisco	[62]
	*Mytilus galloprovincialis*	<LOD–9.39	Adriatic Sea	[56]
	*Ruditapes decussatus*	0.9–13	Portugal	[58]
	*Corbiculidae*	5.0–44	China	[60]
	*Mytilus edulis trossulus*	730 ± 290	Adriatic Sea	[61]
	*Anomalocardia brasiliana*	1.93	Brazil	[57]
	*Mytilus edulis*	1.78	Brazil	[57]
Naproxen	*Geukensia demissa*	<LOQ	San Francisco	[62]
	*Ruditapes decussatus*	1.4–3.9	Portugal	[58]
	*Mytilus edulis trossulus*	473 ± 76	Adriatic Sea	[61]
	*Anomalocardia brasiliana*	<LOQ	Brazil	[57]
	*Mytilus edulis*	<LOQ	Brazil	[57]
Paracetamol	*Mytilus edulis*	≤115	Belgium	[55]
	*Mytilus galloprovincialis*	<LOD	Adriatic Sea	[56]
Piroxicam	*Crassostrea gigas; Chamelea gallina; Mytilus galloprovincialis*	<LOD	Ebro Delta, Spain	[26]
Propyphenazone	*Crassostrea gigas; Chamelea gallina; Mytilus galloprovincialis*	<LOD	Ebro Delta, Spain	[26]

LOD—limit of detection; LOQ—limit of quantification; n.d.—not detected.

**Table 4:** Compilation of different sample preparation and extraction procedures for the analytical determination of NSAIDs in bivalves.

Species	Drugs	NSAIDs	Sample Preparation			Analytical Method	Recovery (%)	References
			Extraction Method	Extraction Procedures	Purification Method			
Blue mussel (*Mytillus edulis*)	5 drugs, including 2 NSAIDs	DIC	PLE	PLE performed on a Dionex ASE 200; extraction solvent: ACN/H_2_O (3:1); 3 cycles of 5 min at 60 °C; extracts evaporated to dryness using N_2_, followed by the addition of H_2_O Milli-Q until 200 mL	SPE	LC-MS/MS	83 ± 8	[23,54]
		MA					104 ± 12	
Pacific oyster (*Crassostrea gigas*)	23 drugs and metabolites, including 9 NSAIDs and metabolites	COD	PLE	PLE performed on a Dionex ASE 350; extraction solvent: MeOH/H_2_O (1:2); 3 cycles of 5 min at 50 °C; extracts evaporated to dryness using N_2_, followed by the addition of H_2_O until 200 mL and then 6 mL of Na_2_EDTA were added	SPE	UHPLC-MS/MS	49.3 ± 2 and 41.7 ± 4.3	[14,26]
		PHE					34.5 ± 3.3 and 42.1 ± 3.9	
		PRO-PHE					45.3 ± 12 and 42.5 ± 3.7	
		PIR					40.1 ± 3.5 and 32.8 ± 2.6	
Mediterranean mussel (*Mytilus galloprovincialis*)		COD					43.1 ± 4.4 and 49.5 ± 2.9	
		PHE					48.2 ± 3.1 and 48.6 ± 3.2	
		PRO-PHE					47.7 ± 0.1 and 50.3 ± 3.6	
		PIR					62.2 ± 2.3 and 63.1 ± 4.4	
Striped venus clam *(Chamelea gallina)*		COD					54 ± 2.8 and 36.1 ± 11.7	
		PHE					56.4 ± 0.3 and 59.3 ± 7.5	
		PRO-PHE					71.7 ± 18 and 68.5 ± 17.4	
		PIR					46.4 ± 12.7 and 43.7 ± 16.3	
Blue mussel *(Mytillus edulis)*	34 drugs and metabolites, including 2 NSAIDs and metabolites	DIC	SLE and centrifugation	Extraction solvent: ACN with 1% formic acid; 6 mL added to 1 g of sample and solution vortexed (5 min, 2500 rpm); centrifugation (10 min, 4500 rpm, 20 °C); scupernatant decanted (in a 15 mL polypropylene tube) and evaporated to dryness; reconstituted in 1mL MeOH/ACN (75:25)	SPE	UHPLC-MS/MS	86 ± 3.4	[22]
		4-OH DIC					61 ± 8.9	
Pacific oyster *(Crassostrea gigas)*		DIC					80 ± 4.2	
		4 OH DIC					46 ± 11.1	
Mediterranean mussel *(Mytilus galloprovincialis)*	7 drugs, including 5 NSAIDs	AS	QuEchERs	Extraction solvent: ACN 10 mL of H_2_O + 1 g of sample, shake for 1 min; 10 mL ACN added and shake 1 min; add the citrate buffer package and shake (15 s manually and 45 s on a vortex); centrifuge (5 min at 7000 rpm); supernatant was transferred to a 15 mL tube with 1 g of silica gel (dispersive sorbent); tube shaken (15 s manually and 45 s in a vortex) and then centrifuged (5 min at 7000 rpm); 1 mL of supernatant was evaporated to dryness (N_2_)	dSPE	LC-MS/MS	61	[78]
		KET					91	
		NAP					95	
		DIC					93	
		IBU					90	
Zebra Mussel (*Dreissena polymorpha*)	NSAIDs	DIC and metabolites	Modified QuEchERs	Extraction solvent: ACN and heptane 5 mL of H_2_O was added to 0.1 g of sample (50 mL tube); 10 mL of ACN and 200 μL of heptane were added to the tube (vortexed for 15 s). The “acetate salt” was added and the tube was shaken (10 s manually, 20 s in a vortex, and 3 min in a centrifuge at 10,000 rpm); 6 mL of supernatant was evaporated to dryness (N_2_)	dSPE	LC-MS/MS	93–105 ± 8–16	[65]
Mediterranean mussel *(Mytilus galloprovincialis)*	7 drugs, including 3 NSAIDs	KET	MAME	Surfactant (3% *v*/*v*) was added to the sample (1g of mussel homogenate) in the container up to 10 mL. MAME extraction followed, and then the extract was filtered with a 0.45 μm nylon membrane before SPE	SPE	LC-UV/DAD	100 ± 4	[3]
		NAP					97 ± 4	
		IBU					106 ± 7	
Blue mussel (*Mytilus edulis*)	14 pesticides, 10 PFCs, and 11 drugs, including 4 NSAIDs	PAR	PLE	PLE performed on a Dionex ASE 350; extraction solvent: ACN/H20 (3:1) with 1% formic acid	SPE	UHPLC-MS/MS	97 ± 26	[55]
		AS					103 ± 10	
		KET					100 ± 12	
		DIC					98 ± 16	
Green Mussel (*Perna viridis*)	7 EDCs, 37 drugs, including 3 NSAIDs	DIC	SLE and centrifugation	Extraction solvent: ACN/MeOH (1:1); 8 mL of the extraction solvent was added to the sample (between 1 and 1.5 g) to a 50 mL tube, which was then shaken (for 16 h at 200 rpm); the tubes were centrifuged (30 min at 12,000 rpm) and the supernatant evaporated to dryness (N_2_)	Sem Purificação	LC-MS/MS	55 ± 5	[70]
		IBU					91 ± 8	
		NAP					41 ± 3	
Venus clam (*Ruditapes decussatus)*	24 drugs, including 3 NSAIDs	DIC	QuEChERS	Extraction solvent: ACN 10 mL of ACN was added to the sample (1 g in a 50 mL tube) and the solution was vortexed (5 min) before being transferred to QuEChERS tubes (with a given combination of salts) which were also vortexed (5 min); samples were then centrifuged (10 min at 4000 rpm)	dSPE	LC-MS/MS	67.2–101.8	[58]
		IBU					104–92.4	
		NAP					67.3–79.2	
Pacific mussel (*Mytilus edulis trossulus*)	3 estrogens and 5 NSAIDs	DIC	PLE	PLE performed on a Dionex ASE 350. Extraction solvent: MeOH:H_2_O (1:1, *v*/*v*), EtOH:H_2_O (1:1 *v*/*v*); ACN:H_2_O (1:1 *v*/*v*). Optimized conditions: preheating for 5 min at 80 °C, 3 min extraction over three static cycles and a pressure of 1500 psi	SPE	GC-MS	61 ± 11	[61]
		NAP					92 ± 6	
		KET					110 ± 9	
		IBU					104 ± 3	
		PAR					66 ± 4	
Mediterranean mussel (*Mytilus galloprovincialis*)	7 drugs, including 3 NSAIDs	AS	PLE	PLE performed on a Dionex ASE 350. Extraction solvent mixture: MeOH:H_2_O (1:1, *v*/*v*), EtOH:H_2_O (1:1 *v*/*v*); ACN:H_2_O (1:1 *v*/*v*). Optimized conditions: preheating for 5 min at 80 °C, 3 min extraction over three static cycles, and a pressure of 1500 psi	SPE	LC-MS/MS	74	[81]
		KET					82	
		NAP					88	
		DIC					82	
		IBU					89	
		OX					90 ± 6	
Mediterranean mussel (*Mytilus galloprovincialis*)	41 drugs, including 3 NSAIDs	PAR	FUSLE followed by filtration	Extraction solvent: MeOH/MilliQ H_2_O (95:5); 0.5 g of the sample was added with the extraction solvent to a 40 mL container and then the FUSLE step occurred for 30 s; extraction took place at 0 °C; the extracts were evaporated to dryness (N_2_)	SPE	LC-MS/MS	112 ± 8	[75]
		DIC					101 ± 8	
		KET					93 ± 10	

ACN—acetonitrile; AS—salicylic acid; CBZ—carbamazepine; CIT—citalopram; COD—codeine; DAD—DIC photodiode detector—diclofenac; dSPE—dispersive solid-phase extraction; EDC—endocrine disruptor; EtOH—ethanol; FLU—fluoxetine; FUSLE—solid–liquid extraction focused on ultrasound; GC—gas chromatography; H_2_O—water; HRMS—high-resolution mass spectrometry; IBU—ibuprofen; KET—ketoprofen; LC—liquid chromatography; LOD—limit of detection; LOQ—limit of quantification; MA—mefenamic acid; MAME–microwave-assisted micellar extraction; MeOH—methanol; MS—mass spectrometry; NAP—naproxen; NOR-FLU—norfluoxetine; NOR-SER—norsertraline; NSAIDs—non-steroidal anti-inflammatory drugs; OX—oxcarbazepine; PAR—paracetamol; PARO—paroxetine; PHE—phenazone; PIR—piroxicam; PLE—pressurized liquid extraction; PRO-PHE—propyphenazone; QuEChERS—quick, easy, cheap, effective, rugged, and safe; DPR—relative standard deviation; SER—sertraline; SLE—solid–liquid extraction; SPE—solid-phase extraction; SSRIs—selective serotonin reuptake inhibitors; UHPLC—ultra-high-performance liquid chromatography; UV—ultraviolet.

**Table 5:** SPE conditions compiled from the revised bibliography for the determination of NSAIDs in bivalves.

Cartridge	Conditioning	Washing	Elution	Reference
Strata-X (6 mL, 200 mg)	6 mL MeOH, 6 mL H_2_O	6 mL H_2_O	3 mL ethyl acetate/acetone (1:1) 3 times	[23,54]
Oasis HLB (6 mL, 200 mg)	6 mL MeOH, 6 mL H_2_O	6 mL H_2_O	6 mL MeOH	[14,26]
Oasis HLB (6 mL, 150 mg)	5 mL MeOH twice 5 mL H_2_O	5 mL H_2_O twice	0.75 mL MeOH twice	[3]
Strata-X (6 mL, 200 mg)	5 mL MeOH 5 mL H_2_O	5 mL H_2_O	3 mL MeOH twice	[55]
Strata-X (3 mL, 200 mg)	3 mL MeOH 3 mL H_2_O	3 mL MeOH 5% in H_2_O 3 mL n-hexane	3 mL MeOH twice	[61]
Oasis MAX (6 mL, 150 mg)	5 mL MeOH 5 mL H_2_O	3 mL H_2_O with 5% NH_4_OH 10 mL MeOH	10 mL MeOH with 5% HCOOH	[81]
Oasis HLB	2 mL MeOH 2 mL H_2_O	-	6 mL MeOH	[76]
Oasis HLB (200 mg)	5 mL MeOH 5 mL H_2_O twice (last at pH = 2)	6 mL H_2_O	6 mL MeOH	[75]

**Table 6:** Compilation of different analytical techniques and conditions in the analytical determination of NSAIDs in bivalves.

Species	Pharmaceuticals	NSAIDs	Analytical Method	Analytical Column	Mobile Phase	Detection	LOD and LOQ	Reference
Blue mussel (*Mytillus edulis*)	5 drugs, including 2 NSAIDs	DIC	LC-MS/MS	Waters Sunfire C18, 150 × 2.1 mm, 3.5 μm	(A) (80:20) 13 mM ammonium acetate in H_2_O/can; (B) ACN 0.3 mL/min	ESI (+/−) ion trap SRM	LOQ: 29 ng/g	[23,54]
		MA					LOQ: 23 ng/g	
Pacific oyster (*Crassostrea gigas*)	23 drugs and metabolites, including 4 NSAIDs	COD	UHPLC-MS/MS	Acquity HSS T3, 50 × 2.1 mm, 1.8 μm (ESI +). Acquity BEH C18 50 × 2.1 mm, 1.7 μm (ESI −)	ESI (+): (A) MeOH; (B) 10 mM formic acid/ammonium, pH 3.2 0.5 mL/min. ESI (−): (A) ACN; (B) 5 mM ammonium acetate/ammonia at pH 8 0.6 mL/min	ESI (+/−) QqLIT SRM	LOD: 0.02 ng/g; LOQ: 0.08 ng/g	[14]
		PHE					LOD: 0.05 ng/g; LOQ: 0.18 ng/g	
		PRO-PHE					LOD: 0.01 ng/g; LOQ: 0.02 ng/g	
		PIR					LOD: 0.03 ng/g; LOQ: 0.11 ng/g	
Mediterranean mussel (*Mytilus galloprovincialis*)		COD					LOD: 0.02 ng/g; LOQ: 0.06 ng/g	
		PHE					LOD: 0.10 ng/g; LOQ: 0.32 ng/g	
		PRO-PHE					LOD: 0.04 ng/g; LOQ: 0.15 ng/g	
		PIR					LOD: 0.06 ng/g; LOQ: 0.20 ng/g	
Striped venus clam *(Chamelea gallina)*		COD					LOD: 0.02 ng/g; LOQ: 0.06 ng/g	
		PHE					LOD: 0.05 ng/g; LOQ: 0.17 ng/g	
		PRO-PHE					LOD: 0.01 ng/g; LOQ: 0.02 ng/g	
		PIR					LOD: 0.04 ng/g; LOQ: 0.13 ng/g	
Blue mussel (*Mytillus edulis*)	34 drugs and metabolites, including 4 NSAIDs	DIC	UHPLC-MS/MS	Luna Omega Polar C18, 100 × 2.1 mm, 1.6 µm (acidic conditions) Waters Acquity BEH C18, 50 × 2.1 mm, 1.7 µm (basic conditions)	Acidic conditions: (A) H_2_O + 0.15% formic acid; (B) (90:10) ACN in H_2_O with 0.15% formic acid 0.5 mL/min. Basic conditions: (A) (95:5) H_2_O in ACN (B) (90:10) ACN in H_2_O 0.5 mL/min	ESI (+/−) QqQ MRM	LOQ: 0.15 ng/g	[22]
		4 OH DIC					LOQ: 1.10 ng/g	
		IBU					-	
		NAP					LOQ: 8.50 ng/g	
Pacific oyster (*Crassostrea gigas*)		DIC					LOQ: 0.10 ng/g	
		4 OH DIC					LOQ: 0.52 ng/g	
		IBU					-	
		NAP					LOQ: 5.62 ng/g	
Mediterranean mussel (*Mytilus galloprovincialis*)	7 drugs, including 5 NSAIDs	AS	LC-MS/MS	Ascentis Express Fused-Core C18, 50 × 4.6 mm, 2.7 μm	(A) 0.5% acetic acid in H_2_O; (B) ACN 0.6 mL/min	ESI (−) QqQ MRM	LOD: 5 ng/g	[78]
		KET					LOD: 50 ng/g	
		NAP					LOD: 2.5 ng/g	
		DIC					LOD: 5ng/g	
		IBU					LOD: 50 ng/g	
Zebra Mussel (*Dreissena polymorpha*)	NSAIDs	DIC and transformation products (metabolites)	LC-MS/MS	Poroshell 120 SB-C8, 50 × 2.1 mm, 2.7 μm	(A) 0.01% formic acid in H_2_O; (B) MeOH 0.6 mL/min	ESI (+) QqQ MRM	LOD: 0.17 ng/g; LOQ: 0.29 ng/g	[65]
Mediterranean mussel (*Mytilus galloprovincialis*)	7 drugs, including 3 NSAIDs	KET	HPLC-UV/DAD	Waters Nova-Pack C18, 150 × 3.9 mm, 4 μm	MeOH in H_2_O (pH 3.0 with acetic acid) 1.0 mL/min	UV	LOD: 0.12 ng/g; LOQ: 0.41 μg/g	[3]
		NAP					LOD: 0.03 μg/g; LOQ: 0.10 μg/g	
		IBU					LOD: 0.06 μg/g; LOQ: 0.21 μg/g	
Blue mussel (*Mytilus edulis*)	14 pesticides, 10 PFCs, and 11 drugs, including 4 NSAIDs	PAR	UHPLC-MS/MS	Nucleodur Pyramid C18, 100 × 2 mm, 1.8 μm	(A) 0.08% formic acid in water; (B) 0.08% formic acid in can; (C) isopropanol	HESI (+) QqQ SRM	LOQ: 2.5 ng/g	[55]
		AS					LOQ: 10 ng/g	
		KET					LOQ: 5 ng/g	
		DIC					LOQ: 2.5 ng/g	
Green mussel (*Perna viridis*)	7 EDCs and 37 drugs, including 3 NSAIDs	DIC	LC-MS/MS	Poroshell 120 SB-C18, 150 × 2.1 mm, 2.7 μm	-	ESI (+/−) QqQ MRM	LOD: 0.24 ng/g	[70]
		IBU					n/a	
		NAP					LOD: 1.4 ng/g	
Venus clam (*Ruditapes decussatus*)	24 drugs, including 3 NSAIDs	DIC	LC-MS/MS	Kinetex EVO C18, 50 × 2.1 mm, 2.6 μm	Acidic conditions: (A) H_2_O + 0.01 mM ammonium acetate + 0.5% formic acid; (B) MeOH 0.3 mL/min. Basic conditions: (A) H_2_O + 0.05 ammonia; (B) MeOH 0.5 mL/min	ESI (+/−) QqQ MRM	LOD: 0.84 ng/g	[58]
		IBU					LOD: 0.6 ng/g	
		NAP					LOD: 0.75 ng/g	
Pacific mussel (*Mytilus edulis trossulus*)	NSAIDs	DIC	GC-MS	Zebron ZB-5MSi, 30 m × 0.25 mm × 0.25 µm	-	-	LOD: 2 ng/g; LOQ: 5 ng/g	[61]
		NAP					LOD: 1 ng/g; LOQ: 4 ng/g	
		KET					LOD: 1 ng/g; LOQ: 4 ng/g	
		IBU					LOD: 1 ng/g; LOQ: 3 ng/g	
		PAR					LOD: 2 ng/g; LOQ: 5 ng/g	
Mediterranean mussel (*Mytilus galloprovincialis*)	7 drugs, including 3 NSAIDs	AS	LC-MS/MS	Ascentis Express C18, 5 cm × 4.6 mm, 2.7 μm	(A) 0.5% ethanoic acid in H_2_O; (B) ACN 0.6 mL/min	ESI (−) QqQ MRM	LOD: 2 ng/g	[81]
		KET					LOD: 50 ng/g	
		NAP					LOD: 2.5 ng/g	
		DIC					LOD: 2.5 ng/g	
		IBU					LOD: 50 ng/g	
Mediterranean mussel (Mytilus galloprovincialis)	41 compounds, including 3 NSAIDs	PAR	LC-MS/MS	Kinetex F5 100 A, 100 × 2.1 mm, 2.6 μm	(A) H_2_O /MeOH (95:5); (B) MeOH/H_2_O (95:5), both with 0.1% methanoic acid 0.3 mL/min	ESI (+/−) QqQ SRM	LOD: 20 ng/g	[75]
		DIC					LOD: 1 ng/g	
		KET					LOD: 2 ng/g	

ACN—acetonitrile; AS—salicylic acid; COD—codeine; DAD—DIC photodiode detector—diclofenac; ESI—electrospray ionization; EtOH—ethanol; GC-MS—gas chromatography coupled to mass spectrometry; H_2_O—water; HESI—heated electrospray ionization; IBU—ibuprofen; KET—ketoprofen; LC-MS/MS—liquid chromatography coupled to tandem mass spectrometry; LOD—limit of detection; LOQ—limit of quantification; MA—mefenamic acid; MeOH—methanol; MRM—multiple reaction monitoring; MS—mass spectrometry; NAP—naproxen; NSAIDs—non-steroidal anti-inflammatory drugs; QqLIT—quadrupole linear IT; QqQ—triple quadrupole; SRM—selected reaction monitoring; UHPLC-MS/MS—ultra-high-performance liquid chromatography coupled to tandem mass spectrometry.

## Data Availability

Not applicable.
